# Suspected Sugammadex Hypersensitivity following Repeated Administration in the Setting of Multiple Flap Takebacks in a Brief Timespan

**DOI:** 10.1155/2021/5716159

**Published:** 2021-08-23

**Authors:** Elizabeth G. Zolper, Alan H. Kim, Kevin G. Kim, Paige K. Dekker, Jenna C. Bekeny, David H. Song, Kenneth L. Fan

**Affiliations:** ^1^Department of Plastic and Reconstructive Surgery, MedStar Georgetown University Hospital, 3800 Reservoir Road Northwest, Washington, DC 20007, USA; ^2^Department of Anesthesiology, MedStar Georgetown University Hospital, 3800 Reservoir Road Northwest, Washington, DC 20007, USA

## Abstract

Sugammadex hypersensitivity is an uncommon event that typically occurs at higher doses. We report a case of suspected sugammadex hypersensitivity in a patient who developed hypoxia and bronchospasm following three administrations of the standard 2 mg/kg doses of sugammadex within 26 hours due to flap takebacks. Hypersensitivity to sugammadex was not initially suspected given that the patient had previous exposures. Diagnoses of pneumothorax, hemothorax, mucus plug, and tracheal tube malposition were immediately ruled out. Furthermore, the onset of hypoxia with sudden loss of tidal volume, development of high peak airway pressures, and temporal correlation with sugammadex administration all supported bronchospasm secondary to a hypersensitivity reaction. Sugammadex is a useful agent for neuromuscular blockade reversal; however, it is critical to carefully examine all adverse reactions. This case report highlights the importance of considering hypersensitivity reactions in the setting of repeat sugammadex administrations in a limited timeframe, such as in free flap reconstruction requiring multiple takebacks to the operating room in the setting of flap compromise.

## 1. Introduction

Sugammadex, a reversal agent for rocuronium bromide- or vecuronium bromide- induced neuromuscular blockade, was approved by the United States Food and Drug Administration (FDA) in 2015. [1] It antagonizes binding of neuromuscular blocking agents to nicotinic cholinergic receptors by forming a complex with the agent [1]. The recommended dose for routine reversal is 2–4 mg/kg but ultimately depends on the depth of the residual neuromuscular blockade [1]. The half-life of sugammadex is approximately two hours [1]. The incidence of sugammadex hypersensitivity is 0.3 to 7% at the recommended doses for routine reversal [2, 3]. We report a case of suspected sugammadex hypersensitivity in a patient undergoing autologous breast reconstruction requiring multiple flap takebacks and repetitive low-dose sugammadex exposures within a twenty-six hour period. The required Institutional Review Board approval and written Health Insurance Portability and Accountability Act (HIPAA) authorization have been obtained. This manuscript adheres to the applicable CARE guidelines (for CAse REports).

## 2. Case Description

A 48-year-old female (92.8 kg) with a history of hypertension, tachycardia, gastroesophageal reflux disease, anemia, and obesity underwent bilateral mastectomy and immediate deep inferior epigastric perforator (DIEP) flap reconstruction performed under general endotracheal tube anesthesia (GETA) with rocuronium. She had previously undergone general anesthesia without complications. The intraoperative course was uncomplicated. Intraoperative fluids targeted a urinary output of 0.5 mL/kg/hr. The estimated blood loss was 250 ml. She received a 250 mg dose of sugammadex for reversal and was extubated without complication.

Twelve hours later, concerns for left flap compromise and hematoma necessitated emergent takeback. Her hematocrit had dropped from 34.7 to 25.6 over five hours, and lactate was elevated to 8.2. She was resuscitated appropriately preoperatively and received two units of packed red blood cells intraoperatively. She underwent GETA with rapid sequence induction (RSI) with succinylcholine and rocuronium for neuromuscular blockade maintenance. After hemostasis was achieved, 200 mg of sugammadex was administered for reversal. Extubation and postanesthesia monitoring proceeded without complication.

Seven hours after initial takeback, the patient returned to the operating room (OR) due to recurrent flap compromise. She had been adequately resuscitated after the second operation. She remained hemodynamically stable and off vasopressors. GETA with RSI with rocuronium was utilized again. The intraoperative course was uncomplicated with minimal blood loss. The patient was on a heparin infusion throughout. Further fluid resuscitation was provided due to reduced urinary output. Initial arterial blood gas (ABG) was stable (T1, Table 1).

During closure, 185.6 mg of sugammadex was administered. About five minutes later, end tidal CO_2_ acutely diminished in amplitude without change in patient positioning. There was a sudden loss in tidal volume (TV) from 350 ml to absent breath with high-pressure alarm. Ventilation was immediately switched from mechanical to manual. Increased inspiratory pressure was required to maintain TV over 200 mL. After dropping to 63%, SpO_2_ recovered to 93% with ventilation at 100% FiO_2_.

Breath sounds were equal but diminished in intensity bilaterally with wheezing. Albuterol was administered via the endotracheal tube (ETT). A suction catheter passed along the length of the ETT did not reveal significant secretions or kinking. The head of the bed was elevated to minimize compressive effects. The patient maintained her saturations on mechanical ventilation with additional positive end-expiratory pressure.

When attempting to wean mechanical ventilation, SpO_2_ dropped again requiring increased peak inspiratory pressures to recover. Blood pressure and heart rate were relatively unchanged. There were no cutaneous changes. ABG revealed low PaO_2_ (T2 Table 1). Ultrasound was unrevealing for pneumothorax. ETT position was unchanged at a depth of 22 cm consistent with prior cases. Portable chest radiograph (CXR) confirmed absence of pneumothorax and ETT positioning. Given the lack of a clear etiology for acute hypoxemia, she remained intubated and was transferred to the intensive care unit (ICU). She passed a spontaneous breathing trial the next morning and was successfully extubated. She was discharged home in stable condition five days later.

## 3. Discussion

In this case, suspected hypersensitivity occurred immediately following administration of a 2–3 mg/kg sugammadex dose. Sugammadex hypersensitivity presenting with acute oxygen desaturation has been previously reported, however decreased SpO_2_ is typically associated with erythema, soft tissue edema, urticaria, or hypotension [4, 5]. Furthermore, hypersensitivity reactions have been reported more frequently at higher doses of sugammadex [2, 3]. Nonetheless, there is no clear dose-dependent relationship, and hypersensitivity has been reported at doses as low as 1.9 mg/kg [5]. Hypersensitivity associated with multiple sugammadex exposures over a short interval and presentation as isolated bronchospasm with hypoxia are both unique (Table 2).

Sugammadex has a half-life of approximately two hours. [1] The preceding dose of sugammadex should have therefore minimally impacted exposure to sugammadex, as the period between the doses exceeded that of four half-lives. Sugammadex is renally cleared; moderate renal impairment can increase exposure by a factor of 2.42 [1, 6]. Our patient's estimated creatinine clearance decreased to 45.12 mL/min after the third operation, corresponding with moderate renal impairment in the study by Min et al. [6]. Renal function may have declined prior to the second and third doses of sugammadex, increasing exposure and resulting in hypersensitivity despite a low administered dose.

Hypersensitivity to sugammadex was not initially suspected as the patient had previous exposures. Differential diagnoses of pneumothorax, hemothorax, mucus plug, and ETT kinking were ruled out first. Postoperative CXR showed the ETT just superior to the carina. This was attributed to patient positioning shifting the ETT towards the carina, as changes in peak airway pressure, TV, or end tidal CO_2_ indicative of an ETT positioned in the mainstem were not observed intraoperatively. The sudden loss of TV, development of high peak airway pressures, temporal correlation with sugammadex administration, and consistent depth of ETT placement all support bronchospasm.

Several factors may have contributed to airway hyperreactivity during the third operation, including prolonged and repeated intubation which could have irritated the airways, increasing susceptibility to subsequent insult. There are reports of bronchospasm secondary to sugammadex in combination with desflurane in patients with undiagnosed underlying pulmonary disease or no history of pulmonary disease [7, 8].

Due to multiple takebacks for flap compromise, sugammadex was administered three times within 25 hours and 25 minutes. Multiple returns to the OR also necessitated repeated intubation and inadvertently led to decreased renal clearance in association with hypovolemia. In this setting, the third, albeit low, dose of sugammadex tipped the proverbial scales into bronchospasm. This is of utmost importance in the setting of free tissue transfer in which takebacks occur in 5.9 to 8.8% of flaps dependent on the site of reconstruction [9, 10]. Although bronchospasm is multifactorial and the contribution of sugammadex is difficult to prove definitively, the impetus remains to reconsider repeated administration of sugammadex in a short timeframe, especially in the setting of recurrent flap takeback or other surgical procedures which may necessitate multiple takeback procedures.

## Figures and Tables

**Table 1:** Arterial blood gases from the third operation.

Labs	T1^*a*^	T2^*b*^	T3^*c*^
pH	7.32	7.20	7.26
pCO_2_ (mmHg)	47.0	54.0	54.0
pO_2_ (mmHg)	157.0	61.0	173.0
Hgb (g/dL)	7.5	7.1	7.1
K^+^ (mmol/L)	3.6	4.1	4.2
Na^+^ (mmol/L)	138	142	141
BE (mmol/L)	−1.9	−6.5	−5.3
Glucose (mmol/L)	129	155	182
Lactate (mmol/L)	0.8	1.3	1.1

^a^T1 is intraoperative during the second takeback, mechanically ventilated with FiO_2_ of 75%. ^b^T2 is obtained at 24 minutes following sugammadex administration and onset of acute hypoxia, manually ventilated with FiO_2_ of 100%. ^c^T3 is postoperative when the patient had stabilized and was admitted to the ICU, mechanically ventilated with FiO_2_ of 100%. BE: base excess.

**Table 2:** Sugammadex dosing.

Sugammadex dose (mg/kg)	Elapsed time from prior dose (hours:minutes)
2.69	N/A
2.15	15 : 56
2.00	9 : 29

The patient (92.8 kg) received a dose of sugammadex during each operation for a total of three doses given over 25 hours and 25 minutes.

## Data Availability

Data are available from the corresponding author upon request, to protect patient privacy.
